# Effects of skin care habits on the development of rosacea: A multi-center retrospective case-control survey in Chinese population

**DOI:** 10.1371/journal.pone.0231078

**Published:** 2020-04-27

**Authors:** Ying-xue Huang, Ji Li, Zhi-xiang Zhao, Bo-lan Zheng, Yu-xuan Deng, Wei Shi, Martin Steinhoff, Hong-fu Xie

**Affiliations:** 1 Department of Dermatology, Xiangya Hospital, Central South University, Changsha, Hunan, China; 2 National Clinical Research Center for Geriatric Disorders, Xiangya Hospital, Central South University, Changsha, Hunan, China; 3 Key Laboratory of Organ injury, Aging and Regenerative Medicine of Hunan Province, Changsha, Hunan, China; 4 Department of Dermatology, University College Dublin, Dublin, Ireland; 5 Department of Dermatology, Hamad Medical Hospital, Weill Cornell University-Qatar, Doha, Qatar; University of California Los Angeles, UNITED STATES

## Abstract

**Background:**

Certain cosmetic habits may trigger or aggravate rosacea, while there is little published epidemiologic evidence to support this point.

**Purpose:**

To examine if daily skin care habits have an effect on the development of rosacea in Chinese population.

**Methods:**

A multi-center retrospective case-control survey of 1,245 rosacea cases and 1,538 skin-healthy controls was conducted in China. Participants completed the questionnaire comprised of demographic characteristics, socioeconomic data and daily skin care habits. Data were collected retrospectively and analyzed using the chi-square test and t-test. Multivariate logistic regression analyses were used to predict rosacea.

**Results:**

The multivariate logistic regression analysis highlighted some results: Dry, oily or mixed skin (OR = 6.3–6.9, *P*< .001), the usage of foaming cleanser (OR = 1.45, 95%CI 1.115–1.886, *P* = .01), make up more than 6 times a week (OR = 2.839, 95%CI 1.962–4.108, *P*< .001), using facial mask more than 4 times a week (OR = 2.56–3.069, *P*< .001), facial treatments at beauty salon more than once a week (OR = 4.946, 95%CI 2.005–12.198, *P =* .0018) and using beauty salon products (OR = 2.334, 95%CI 1.435–3.976, *P* = .0018) are positively correlated with the development of rosacea. Using of moisturizing products (OR = 0.602, 95%CI 0.386–0.983, *P* = .035) and sunscreen cream (OR = 0.303–0.507, *P*< .001 or *P* = .0167 for different frequency) presented significantly negative correlations with rosacea. Frequency of cleansing showed a nonlinear association with rosacea: using facial cleansers 1~3 times per week (OR = 0.647, 95%CI 0.429–0.975, *P*  =  .038) showed beneficial effects while using facial cleanser excessively (twice or more daily) (OR = 2.131, 95%CI 1.394–3.256, *P*< .001) positively correlated to rosacea strongly.

**Conclusions:**

Excessive use of facial cleanser (twice or more a day) and facial mask (more than 4 times a week), frequent makeup (more than 6 times a week), regular skin care in beauty salon (more than once a week), and using beauty salon products were closely correlated to the development of rosacea in Chinese population.

## Introduction

Rosacea is a chronic inflammatory cutaneous disorder that displays a broad diversity of clinical manifestations including central facial erythema which worsens suddenly (flushing), papules and pustules, telangiectasias and hypersensitive symptoms such as itching, stinging, burning, etc.[1]. The current classification system for rosacea describes four distinct clinical subtypes based on the main clinical characters: erythematotelangiectatic rosacea(ETR), papulopustular rosacea(PPR), phymatous rosacea(PhR), and ocular rosacea[2].

Previous studies have demonstrated that the disorders of the innate immune system, dysfunction of facial vascular regulation, neurogenic inflammation, skin barrier destruction, and elevated levels of Demodex mites as risk factors of rosacea[3–8]. Considering the hypersensitivity and hyperactivity of the skin in rosacea patients, skin barrier destruction as a predisposing factor has attracted more and more attention in recent years[6, 9–14]. Fortunately, the destruction can be mitigated by suitable skin care practice[15]. Skin care is a part of the lifestyle that mainly includes skin cleansing, moisturizing, sun protection, and makeup as well[16, 17]. Mild cleansing, moisturizing, and photo protecting were common suggestions of skin care for people with rosacea[15]. However, it has been reported that some inappropriate skin care regimens and specific cosmetic formulations may trigger or aggravate rosacea[16, 18, 19]. During our clinic work, we also noticed that some skin care practice such as excessive facial cleansing and using products from beauty salons could trigger the flare-up of rosacea. On the other hand, patients with rosacea always tended to visit the beauty parlors more or preferred using facial masks. Previous studies have paid more attention to the therapeutic effect of skin care habits/products on rosacea[16, 20–23]. However, there is little published epidemiologic evidence to support the point that skin care habits may affect rosacea occurring. To examine if skin care practice people routinely followed really has protective or accelerating effects on rosacea attack, we conducted a questionnaire-based multi-center case-control survey in a large population in China. Confirming the linkage may aid the dermatologists in providing useful suggestions about skin care regimen to the public to facilitate the prevention and management of rosacea.

## Materials and methods

### Study design

A retrospective case-control survey of 1,308 rosacea cases and 1,560 skin-healthy controls were conducted. All the patients were the first visit to the hospital and met the diagnostic criteria of rosacea based on the National Rosacea Society Expert Committee[24], and diagnosed independently by two board-certified dermatologists. Patients have other concurrent facial skin diseases such as acne, Systemic Lupus Erythematosus, eczema, seborrheic dermatitis, and those who have been accepting treatments were excluded. Age- and sex-matched controls were skin-healthy individuals from the medical examination center who had neither facial skin disease nor familial antecedents of rosacea. All the patients and the healthy controls provided written informed consent. Data were collected from 5 hospitals in 5 cities of China, including Xiangya Hospital of Central South University, the First People’s Hospital of Changde, the First People’s Hospital of Wuhan, Huashan Hospital of Fudan University and Dermatology Hospital of Guangzhou between June 2013 and March 2016. Participants who incompletely answered the questionnaire or healthy control with sensitive skin were excluded. The detailed protocol of this study was approved by the Institutional Review Board of the above five hospitals.

### Survey questionnaires

A self-reported questionnaire mainly including the skin care habits was developed by dermatological experts according to related articles and combined with the skin care habits routinely followed by the Chinese. The sample of the questionnaire was provided in supporting information, which covered age, gender, education level (high school level or less; university or junior college level; master degree or above), monthly income (less than 1000RMB; 1000-2000RMB; 2001-4000RMB, more than 4000RMB), and skin care habits in the previous two-year before the onset of rosacea for the patient group and in the past two years for the control group. The investigated skin habits included skin cleansing, moisturizing, sun protection, makeup, facial mask, beauty salon items, and the sources of skin care or cosmetic products. It was developed based on the four classic steps of skin care combined with some specific skin care habits, which showed a close association with the flare-up of rosacea during our clinic work. Standard trained interviewers completed the questionnaire-based survey. Skin type (normal, dry, oily, and mixed types) and medical records (main characteristics and subtypes of rosacea) were evaluated by a designed dermatologist. Given that there is no consensus on a standardized quantitative method to measure a reliable cutaneous sebum level, skin type of the participants were determined to oily, dry according to the clinical signs such as natural unctuosity, sebaceous follicle dilatation and desquamation as the previous study did[25]. As the sebum release rate of the skin is variable among different anatomic locations of the facial skin and diverse circumstances, a combination of a greasy T-zone and dry cheeks was considered as mixed skin; those without the evident feature of oily or dry were determined as neutral skin.

**Online Resource 1** The sample of the questionnaire.

### Statistical analysis

Continuous variables were described using means and standard deviations, and the differences were tested using the Student t-test. Categorical variables were described using proportion, and differences were tested using the chi-square test for the univariate analysis. Multiple logistic regression analysis was used to estimate the effect size of risk / protective factors for rosacea. The p-value was adjusted by Bonferroni correction to decrease the risk of false-positive results. Odds ratio (OR) was used to indicate the effect size, and the 95% confidence interval (CI) of OR was estimated. For unordered categorical variables (skin type) as well as graded variable, OR for each category was estimated respectively. Interactions between significant factors identified by logistic regression were further modeled. In subgroup analyses, samples were stratified by different skin types. Statistical analyses were performed in SPSS 19.0 (SPSS Inc., Chicago, IL, USA). The significance level for all statistical tests was 0.05, and the hypothesis tests were 2-sided.

## Results

### Characteristics of the study population

A total of 1,245 patients and 1538 controls with informed consent and completed questionnaires were finally enrolled in the study. The major part (98%) of the study population was ethnic Han Chinese. The demographic characteristics of the case and the control group were shown in Table 1. The mean education level of the case group was lower than the control group. There were no statistically differences in gender and income between the case and the control group.

**Table 1:** Comparisons of demographic characteristics between the case and control groups.

Characteristic		Rosacea Cases n (%)	Controls n (%)	*p* value
**Gender**				
	Male	121(9.7)	150(9.8)	0.976
	Female	1124(90.3)	1388(90.2)	
**Age, y**		33.3 ± 10.8	29.5 ± 9.4	<0.001
**Education level**					
	High school level or less	549(43.8)	331(21.5)	<0.001
	University or junior college	653(52.1)	846(55)	
	Master degree or above	52(4.1)	361(23.5)	
**Personal monthly income**					
	<1,000 Yuan	341(27.2)	245(15.9)	0.368
	1,000–2,000 Yuan	110(8.8)	529(34.4)	
	2001–4000 Yuan	295(23.5)	284(18.5)	
	>4000 Yuan	508(40.5)	480(31.2)	
**Working place**				
Outdoor		121(9.7)	117(7.6)	0.048
Indoor		1124(90.3)	1421(92.4)	

(N = 2783).

### Effects of skincare habits on rosacea

A total of 13 factors related to skin care behaviors were evaluated by comparing the rosacea group with the skin-healthy control group through the univariate analysis (Table 2), and the factors which significantly associated with rosacea were further evaluated for multivariate analysis.

**Table 2:** Comparisons of skin care habits between the case and control groups.

		Rosacea N(%)	Control N(%)	*p*-value
Skin type	Neutral	110 (8.8)	529 (34.4)	<0.001
	Dry	339 (27.2)	244 (15.9)	
	Oily	292 (23.5)	284 (18.5)	
	Mixed	504 (40.5)	481 (31.3)	
Skin care frequency	Hardly	241 (19.4)	279 (18.1)	<0.001
	1 / day	220 (17.7)	365 (23.7)	
	2 / day	757 (60.8)	828 (53.8)	
	≥ 3 / day	27 (2.2)	66 (4.3)	
Function of skin care products	Moisturizing	884 (71.0)	1162 (75.6)	0.007
	Whitening	261 (21.0)	368 (23.9)	0.063
	Antiaging	125 (10.0)	195 (12.7)	0.030
	Oil-control	254 (20.4)	236 (15.3)	<0.001
	Antiallergy	200 (16.1)	169 (11)	<0.001
Ways of buying products	Shopping mall/pharmacy	783 (62.9)	925 (60.1)	0.139
	Salon	140 (11.2)	72 (4.7)	<0.001
	Online	142 (11.4)	348 (22.6)	<0.001
	Self-made	24 (1.9)	23(1.5)	0.379
	Direct selling	92 (8)	182 (11.8)	<0.001
Cleansing frequency	Hardly	369 (29.6)	411 (26.7)	<0.001
	1~3 / week	143 (11.5)	357 (23.2)	
	1 / day	429 (34.5)	582 (37.8)	
	≥ 2 / day	304 (24.4)	188 (12.2)	
Type of cleansers	Soap	49 (3.9)	106 (6.9)	0.001
	Foam	497 (39.9)	516 (33.6)	0.001
	Emulsion	216 (17.4)	248 (16.1)	0.389
	Exfoliator	241 (19.4)	285 (18.5)	0.580
Facial mask frequency	Hardly	589 (47.3)	775 (50.4)	<0.001
	1 / week	191 (15.3)	377 (24.5)	
	2~3 / week	259 (20.8)	261 (17.0)	
	4~5/ week	83 (6.7)	37 (2.4)	
	≥ 6/week	123(9.9)	88 (5.7)	
Beauty salon frequency	Hardly	944 (75.8)	1289 (83.8)	<0.001
	≤ 2 / month	156 (12.5)	197 (12.8)	
	3~4 / month	76 (6.1)	40 (2.6)	
	> 1 / week	69 (5.6)	12 (0.8)	
Type of skin care in the salon	Moisturizing	224 (18.0)	155 (10.1)	<0.001
	Whitening	40 (3.2)	68 (4.4)	0.101
	Antiaging	18 (1.4)	49 (3.2)	0.003
	Oil-control	65 (5.2)	30 (2.0)	<0.001
	Antiallergy	50 (4.0)	31 (2.0)	0.002
	Exfoliator	25 (2.0)	16 (1.0)	0.035
	Other	14(1.1)	4(0.3)	0.005
Make-up frequency	Hardly	786 (63.1)	988 (64.2)	<0.001
	1~2 / week	64 (5.1)	211 (13.7)	
	3~5 / week	135 (10.8)	201 (13.1)	
	≥ 6 / week	260 (20.9)	140 (9.1)	
Use foundation products	No	812 (65.2)	1042 (67.7)	0.182
	Yes	433 (34.8)	498 (32.4)	
Use make-up remover after making up	No	930 (74.7)	1118 (72.7)	0.232
	Yes	315 (25.3)	420 (27.3)	
Sunscreen cream frequency	Hardly	880 (70.7)	848 (55.1)	<0.001
	1~2 / week	125 (10.0)	247 (16.1)	
	3~5 / week	67 (5.4)	116 (7.5)	
	≥ 6 / week	173 (13.9)	327 (21.3)	

Sex, age, education level, income, and working place were adjusted with a multiple logistic regression model. The results were shown in Table 2. It produced some potential risk factors that were significantly correlated with rosacea (adjusted *P*<0.05): skin type (dry, oily, or mixed), the usage of foaming cleanser and anti-allergy products, using facial mask frequently (more than 4 times a week), make up more than 6 times a week, using beauty salon products, frequent facial treatments at beauty salon (more than once a week), and accepting oil-control projects in beauty salon. Frequency of cleansing showed a nonlinear association with rosacea: compared with people hardly use facial cleanser, those who used facial cleansers 1~3 times per week were negatively correlated with rosacea (OR = 0.647, 95%CI 0.429–0.975, *P*  =  .038); in contrast, those used facial cleansers excessively (twice or more daily) were closely related to rosacea with an OR of 2.5 (OR = 0.647, 95%CI 0.429–0.975, *P*  =  .038). Significant protective effects were found for applying moisturizing products and sunscreen cream, using anti-aging products, and using skin care products purchased through direct selling or online shopping.

When considering skin type, we found the associations of excessive cleansing (applying facial cleansers twice or more daily) and frequent make up (almost every day) with rosacea were more distinct in those with dry or mixed skin and the OR values were the highest for dry skin. On the other hand, the beneficial effects of using moisturizing cream were only significant for those with dry skin, but insignificant for those with other skin types. Using sunscreen cream was the only factor that showed significant for all skin types **(**Table 3**)**.

**Table 3:** Associations of rosacea with skin care behaviors and subgroup analyses.

	Total patients		Neutral skin		Dry skin		Oily skin		Mixed skin	
	OR (95%CI)	*P* (adjusted *P*^a^)	OR (95%CI)	*P*	OR (95%CI)	*P*	OR (95%CI)	*P*	OR (95%CI)	*P*
**Skin type**										
Neutral	1									
Dry	**6.751 (4.606–9.895)**	**<0.001 (<0.001)**	N/A		N/A		N/A		N/A	
Oily	**6.109 (4.153–8.986)**	**<0.001 (<0.001)**	N/A		N/A		N/A		N/A	
Mixed	**6.624 (4.629–9.480)**	**<0.001 (<0.001)**	N/A		N/A		N/A		N/A	
**Skin care frequency**										
Hardly	1				1		1		1	
1 / day	0.982 (0.567–1.7)	0.947 (0.947)	N/A^b^		**23.417 (1.664–329.48)**	**0.019**	0.548 (0.191–1.573)	0.264	1.360 (0.518–3.572)	0.533
2 / day	**1.841 (1.052–3.221)**	**0.032 (0.043)**	N/A^b^		**27.225 (1.915–387.025)**	**0.015**	1.825 (0.63–5.289)	0.268	2.303 (0.839–6.319)	0.105
≥ 3 / day	0.492 (0.202–1.194)	0.117 (0.138)	N/A^b^		**32.103 (1.571–656)**	**0.024**	0.780 (0.128–4.737)	0.787	0.234 (0.045–1.218)	0.084
**Function of skin care products**										
Moisturizing	**0.602 (0.386–0.938)**	**0.025 (0.035)**	N/A^b^		**0.019 (0.002–0.237)**	**0.002**	0.631 (0.284–1.399)	0.257	0.674 (0.322–1.41)	0.295
Anti-aging	**0.607 (0.396–0.929)**	**0.022 (0.032)**	0.817 (0.206–3.237)	0.773	0.803 (0.265–2.44)	0.699	0.325 (0.092–1.142)	0.080	0.569 (0.292–1.109)	0.098
Anti-allergy	**1.902 (1.297–2.788)**	**0.001 (0.002)**	1.359 (0.338–5.458)	0.666	1.479 (0.477–4.585)	0.498	1.880 (0.801–4.41)	0.147	**2.537 (1.417–4.534)**	**0.002**
**Ways of buying products**										
Salon	**2.334 (1.435–3.796)**	**0.001 (0.002)**	**6.250 (1.589–24.585)**	**0.009**	**4.437 (1.497–13.151)**	**0.007**	0.825 (0.168–4.056)	0.813	**2.222 (1.016–4.86)**	**0.046**
Online	**0.551 (0.391–0.777)**	**0.001 (0.002)**	0.278 (0.074–1.043)	0.058	**0.231 (0.074–0.724)**	**0.012**	**0.408 (0.179–0.927)**	**0.032**	0.821 (0.5–1.347)	0.435
Direct selling	**0.516 (0.339–0.785)**	**0.002 (0.003)**	0.457 (0.118–1.771)	0.257	0.636 (0.239–1.692)	0.365	**0.237 (0.083–0.677)**	**0.007**	0.621 (0.302–1.274)	0.195
**Cleansing frequency**										
Hardly	1		1		1		1		1	
1~3 / week	**0.647 (0.429–0.975)**	**0.038 (0.049)**	**0.240 (0.058–0.996)**	**.049**	0.576 (0.204–1.626)	0.298	**0.361 (0.148–0.882)**	**0.025**	0.887 (0.445–1.768)	0.733
1 / day	1.117 (0.783–1.592)	0.542 (0.556)	1.358 (0.443–4.16)	.592	1.684 (0.721–3.932)	0.228	0.706 (0.328–1.52)	0.374	1.023 (0.553–1.895)	0.942
≥ 2 / day	**2.131 (1.394–3.256)**	**<0.001 (<0.001)**	2.472 (0.682–8.956)	.168	**3.103 (1.110–8.677)**	**0.031**	1.493 (0.617–3.614)	0.374	**2.308 (1.102–4.835)**	**0.027**
**Type of cleansers**										
Foam	**1.450 (1.115–1.886)**	**0.006 (0.01)**	**3.752 (1.54–9.142)**	**0.004**	0.918 (0.457–1.84)	0.809	**2.255 (1.221–4.164)**	**0.009**	1.12 (0.741–1.692)	0.59
**Facial mask frequency**										
Hardly	1		1		1		1		1	
1 / week	0.874 (0.62–1.231)	0.440 (0.477)	1.166 (0.4–3.396)	0.779	0.977 (0.4–2.388)	0.959	1.153 (0.528–2.514)	0.721	0.675 (0.387–1.176)	0.165
2~3 / week	1.460 (1.016–2.096)	0.041 (0.051)	0.494 (0.131–1.86)	0.297	1.733 (0.699–4.298)	0.235	2.267 (0.944–5.447)	0.067	1.504 (0.865–2.615)	0.148
4~5/ week	**3.069 (1.636–5.757)**	**<0.001 (<0.001)**	4.161 (0.528–32.777)	0.176	**23.001 (1.338–395.484)**	**0.031**	**10.250 (2.567–40.926)**	**0.001**	1.644 (0.648–4.17)	0.295
≥ 6/week	**2.560 (1.561–4.198)**	**<0.001 (<0.001)**	2.726 (0.458–16.231)	0.271	**6.826 (1.774–26.263)**	**0.005**	2.572 (0.804–8.223)	0.111	1.968 (0.93–4.166)	0.077
**Beauty salon frequency**										
Hardly	1		1		1		1		1	
≤ 2 / month	0.779 (0.532–1.142)	0.201 (0.231)	1.110 (0.322–3.823)	0.869	0.751 (0.317–1.781)	.516	0.481 (0.158–1.467)	0.199	0.731 (0.407–1.31)	.292
3~4 / month	1.929 (0.989–3.763)	0.054 (0.066)	0.523 (0.056–4.865)	0.569	1.025 (0.172–6.098)	.978	4.091 (0.474–35.342)	0.200	**3.454 (1.256–9.5)**	**0.016**
> 1 / week	**4.946 (2.005–12.198)**	**0.001 (0.002)**	**25.757 (1.902–348.864)**	**0.015**	5.502 (0.536–56.471)	.151	4.657 (0.442–49.076)	0.200	2.703 (0.638–11.452)	.177
**Type of skin care in salon**										
Anti-aging	**0.199 (0.077–0.514)**	**0.001 (0.002)**	N/A^b^		0.368 (0.04–3.419)	0.380	**0.081 (0.009–0.751)**	**.027**	0.229 (0.048–1.082)	.063
Oil-control	**2.554 (1.241–5.256)**	**0.011 (0.017)**	2.510 (0.114–55.432)	0.56	7.866 (0.361–171.275)	0.189	**7.149 (1.209–42.273)**	**.030**	1.911 (1.692–5.278)	.211
**Frequency of make up**										
Hardly	1		1		1		1		1	
1~2 / week	0.813 (0.501–1.32)	0.403 (0.45)	0.320 (0.042–2.451)	0.273	0.914 (0.297–2.818)	.876	0.613 (0.181–2.077)	.432	0.874 (0.425–1.796)	.0714
3~5 / week	1.193 (0.718–1.938)	0.495 (0.522)	1.027 (0.217–4.861)	0.974	0.159 (0.022–1.179)	.072	0.585 (0.155–2.207)	.429	2.520 (1.221–5.199)	0.012
≥ 6 / week	**2.839 (1.962–4.018)**	**<0.001 (<0.001)**	2.422 (0.723–8.114)	0.152	**5.200 (2.073–13.048)**	**<0.001**	1.331 (0.552–3.208)	.524	**3.015 (1.651–5.508)**	**<0.001**
**Frequency of using sunscreen cream**										
Hardly	1		1		1		1		1	
1~2 / week	**0.507 (0.353–0.727)**	**<0.001 (<0.001)**	0.235 (0.044–1.253)	.090	0.712 (0.303–1.673)	0.436	0.622 (0.277–1.394)	0.249	**0.297 (0.165–0.534)**	**<0.001**
3~5 / week	**0.533 (0.328–0.867)**	**0.011 (0.017)**	**0.067 (0.008–0.595)**	**.015**	0.842 (0.257–2.76)	0.776	**0.229 (0.057–0.922)**	**0.038**	0.498 (0.241–1.03)	0.060
≥ 6 / week	**0.303 (0.209–0.44)**	**<0.001 (<0.001)**	0.343 (0.097–1.21)	.096	**0.232 (0.085–0.632)**	**0.004**	**0.391 (0.163–0.934)**	**0.035**	**0.252 (0.144–0.442)**	**<0.001**

^a^The p-value was adjusted by Bonferroni correction.

^b^ The number of these cases was so small that not fit for calculating the OR value.

**Fig 1:**
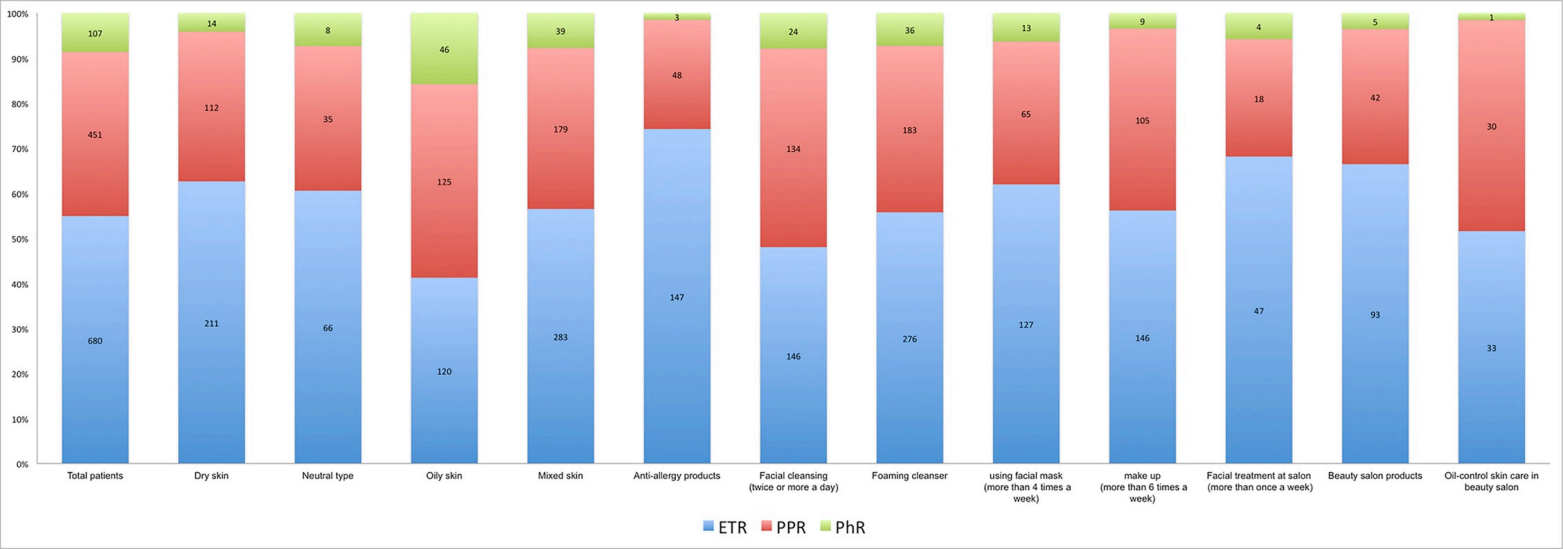
The proportion of the subtypes of ETR, PPR, PhR in groups of rosacea patients with different high-risk factors. ETR was the most dominant subtype in the group of patients using anti-allergy products, PPR accounted for the relative highest proportion in the group of patients doing oil-control skin care in the beauty salon, and the relative proportion of PhR in the group of patients with oily skin was the highest.

Among the 1245 patients with rosacea, there were 680 cases of ETR, 451 cases of PPR, 107 cases of PhR, and 7 cases of ocular rosacea. The distribution of the subtypes of ETR, PPR, PhR among rosacea patients with each possible high-risk factor was shown in Fig 1. Compared with the whole constituent ratio of the total patients, the relative proportion of ETR among patients with dry skin, patients using anti-allergy products or beauty salon products, and patients using facial mask or going beauty salon frequently were much higher; PPR accounted for a relatively higher proportion in patients with oily skin, and patients using facial cleanser frequently or doing oil-control skin care in beauty salon; the relative proportion of PhR in patients with oily skin was the highest among that in patients with other factors.

### Interactive effects

Among significant factors identified by logistic regression, several pairs of potential interactions were considered. The previous risk effects of using foaming cleanser turned beneficial when it interacted with using moisturizers (OR = 0.298, 95% CI 0.16–0.557; *P*< .001). Similarly, the association of using facial masks (more than 4 times a week) with rosacea became insignificant when considering the interaction between using facial masks and purchasing skincare products from shopping mall/pharmacy.

## Discussion

In the current study, we investigated the skin care habits before the onset of the disease in rosacea patients with different skin types and found some skincare behaviors were closely associated with the development of rosacea in Chinese population. As far as we know, this is the first study to investigate the relationship between skin care habits and rosacea occurring.

The oily or dry nature of skin is an essential consideration for skin care. Our results showed a close association of dry, oily, and mixed skin type with rosacea. A predominant oily skin always means abundant sebaceous secretion and high sebum casual levels. As rosacea’s predilection for sebaceous locations, and the efficacy of oral isotretinoin by the mechanism of reducing sebaceous gland size and decreasing sebum production, it is easy to consider rosacea to occur more easily on oily skin. Previous studies also have established that the sebum secretion and sebaceous lipids of the oily skin played an important role in the activation of the immunity mechanism[4, 26]. We also have demonstrated previously that rosacea patients with lesions mainly on the nose had higher oil content on the nasal skin[11], which could explain the higher proportion of PhR in patients with oily skin. Besides, Demodex infestation, a known aggravating factor of inflammation in rosacea, is common in more seborrheic skin[25, 27], which may also contribute to the close relationship of oily skin with rosacea. Unexpectedly, patients with rosacea frequently complain of dryness and tight feeling rather than greasy skin. Previous studies have demonstrated that patients with rosacea had reduced skin surface hydration levels and abnormal sebum composition because of the dysfunctions of skin barrier [11, 13], which might be the main reason for the positive correlation of dry skin with rosacea. Similarly, despite a relatively lower risk, patients with dry skin were also more likely to be infested with Demodex [27]. Another result we found was that the cases of ETR were the largest in patients with dry skin. It would be interesting to investigate the clinical features of different skin types. Based on our results, as dry, oily, and mixed skin were all related to rosacea we concluded that the occurrence of rosacea might not be necessarily linked to a skin type, but is closely related to the imbalance of hydration and sebum level of the skin. Thus it was particularly vital to recommend a daily moisturizing routine and sebum-modifying treatments as adjuvant therapy or prevention for this disorder.

Skin cleansing is the first step of general skin care. In our multivariate analysis, excessive cleansing (using facial cleansers twice or more a day) and the usage of foaming cleanser were closely related to rosacea occurring among Chinese population. Excessive cleansing may cause mechanical damage to the tightly arranged stratum corneum of the epidermis, and bring chemical irritation to the water and lipid-coated membrane of the skin, which will alter the normal pH of our skin[20]. Notably, we found that the proportion of PPR was the highest in patients who have a situation of excessive cleansing, but the reason was so far unknown. Foaming cleansers often contained special surfactants and showed excellent foaming and lathering characteristics, but they may strip natural moisturizing factors from the skin or protective lipids and proteins from the stratum corneum[18], leading to after-wash tightness, dryness and barrier damage. Excessive cleansing and deep cleansing foam might give adverse effects to rosacea by aggravating skin barrier destruction. Although the standard recommendation of facial cleansing for rosacea was to wash the face twice daily[28], based on our results, use facial cleanser less than two times a day would be safer, especially for those with dry skin, as the association was the most distinct. What is more, the foaming cleanser should be avoided.

Moisturizers are the most effective products to maintain and restore the skin barrier[29]. Using moisturizing products showed beneficial effects on rosacea, and the effect was most distinct for dry skin individuals. Also, it could weaken the association of using foaming cleanser with rosacea based on the results of interactive effects. Previous researches have shown moisturizing cream could repair epidermal barrier dysfunction by improving the water content of the skin, reducing the damage to stratum corneum proteins, and maintaining the epidermal lipids, contributing to the patients with rosacea and alleviating the skin barrier dysfunction[21, 30, 31]. Nowadays, bioactive ingredients with different mechanisms are added to the cosmetics to endow them with additional oil-control, anti-aging, brightening, or anti-allergic properties, including exfoliants, natural and herbal ingredients, different kinds of vitamins, proteins, minerals, and other materials[32]. Some of the bioactive ingredients, such as green tea, could display anti-inflammatory or antioxidant activity, thus were considered beneficial for inflammatory skin disorders, including rosacea[33]. But the combination of different categories of ingredients would increase the possibility of skin sensitization and aggravate skin barrier destruction. Our study showed a higher frequency of previously using anti-allergy products in rosacea patients. That might be a result of the disease, as patients with rosacea often suffered sensitive symptoms before the onset of the disease (commonly-termed “pre-rosacea” or “early-onset rosacea”)[3], and they might seek anti-allergy products to relieve the symptom. The high proportion of ETR among the patients using anti-allergy products might be explained by the close relationship of this subtype with the skin barrier. For those products claiming anti-aging, oil-control, or brightening properties, there was no evidence to support that they had positive relationships with rosacea in our study, but doing oil-control skincare in the beauty salon seemed to relate to rosacea closely. It has been reported that salicylic acid and astringent compounds for the sebum-regulating purpose would cause dryness, skin irritation and barrier dysfunction[28]. Therefore, for better prevention and management of symptoms of rosacea, multi-bioactive ingredients and complicated properties were not recommended. The skin care regimen should be as simple as possible, choosing moisturizers without fragrance, antiseptics, and surfactants, just as previous paper suggested[28].

Strict sun protection is strongly recommended to prevent UV-induced rosacea, as UV irradiation is widely acknowledged as triggers for flushing events[7]. We proved the use of sunscreen cream to be a beneficial factor for rosacea as expected, and the effect was significant for all skin types. Notably, other studies have proposed that due to the possible irritants or allergens contained in the sunscreen cream, the irritancy of sunscreen products were easy to occur[34, 35]. Therefore, daily use of sunscreen cream is necessary, but preference should be given to very high tolerance sunscreen products, such as physical sun-blocking creams[28], with simple formulations that contain the smallest possible number of ingredients.

Makeup has been considered to increase the frequency of sensitization because of the mineral oil added in cosmetics. Also, because the thick and occlusive characters contained in cosmetic products were difficult to remove, excessive cleansing was inevitable. In our study, we found frequent makeup (almost every day) had a close relationship with the development of rosacea, and the effect was most distinct for people with dry skin. However, this did not mean that makeup should be avoided. Makeup in moderation and use of foundation products and make-up remover were proved not to increase the risk of rosacea in our study. Corrective makeup and using foundation products can improve skin tone, even skin color, and cover redness, hence increasing the self-confidence of the individuals and exerting decompressing effects, thereby bringing benefits on rosacea[36]. Considering the facial skin of rosacea is always appearance-impaired, foundation products such as green-tinted makeup can be recommended to relieve the cosmetic symptoms of rosacea or other skin disorders[36].

In addition to the everyday skin care habits of using cleansers, moisturizers, and sunscreen products, we found some additional factors that may be associated with the development of rosacea in the Chinese patients. These factors included frequent use of facial masks, regularly visiting a beauty salon, and using beauty salon products. Applying a facial mask is very prevalent because of its instant hydrating function, covering nearly 50% of the participants in the current study. It is expected that the applied mask would moisturize the skin properly and deeply, remove the sebum, and rejuvenate the skin. Interestingly, it was proved to be no benefits but associated with the development of rosacea in our study. Although there were concerns about the safety of facial masks, for example, the different artificial fragrances and dyes, parabens, and phthalate esters used for masks could be harmful to the skin, and the number of bacteria on the surface skin might be increased when applying a mask, there was no evidence about the association of using facial masks with rosacea at present. Based on our results of the interaction effect, the association turned insignificant when facial mask interacted with purchasing cosmetics from shopping mall/pharmacy. Thus we inferred the positive correlation might be explained by the current non-standard cosmetic market and the inadequate supervision of cosmetic manufacturing in our country, that many unqualified and unregulated skin care products escape into the marketplace. According to the National Food and Drug Administration website of China, from 2016 to 2018, glucocorticoids were illegally added to many batches of cosmetic products. Notably, the abuse of glucocorticoid was particularly prevalent in facial masks and beauty salon products for short-term commercial effects[37, 38]. It has well accepted that dilation of blood vessels, red spots, and thinning of the facial skin like the feature of ETR would take place in the process of long-term use of glucocorticoid[38]. Therefore, if the source and the contents of skin care products are unreliable, the more frequent and more regular skin care people do, the more likely they will suffer skin barrier damage and even attack steroid-induced rosacea. That could also explain the relatively high proportion of ETR among the patients using facial masks or beauty salon products, and patients going beauty salons frequently. Based on these results, choosing safe and reliable steroid-sparing products is a crucial precondition for skin care.

In this investigation, we found a lower level of education in rosacea patients, which was consistent with the results of our previous study[39]. Lower education means less knowledge of avoiding trigger factors, so they are more likely to suffer a flare-up of rosacea. Suffering rosacea might affect the work productivity of the patients and then influence the income levels more or less[40], but there were multiple factors influencing income, so we did not found a significant correlation of income levels with rosacea based on the multiple logistic regression.

## Limitations

As the preliminary reports, our study has limitations in terms of the area and size of the survey. First, participants did not cover the whole Chinese population. Secondly, the present study was a retrospective case-control research, so the strength of cause and effect linkage was not strong enough. However, our study may be the first epidemiologic study showing skin care habits and their correlation with rosacea, and is valuable for establishing skincare guidelines for rosacea, but a well-designed prospective study is needed.

## Conclusions

Poor skin care habits, including excessive use of facial cleanser (twice or more a day) and everyday makeup (more than 6 times a week) were correlated closely to the development of rosacea. Using mild moisturizers and sunscreen cream presented beneficial effects on rosacea. Mild cleansing (using facial cleanser less than two times a day and avoiding foaming cleanser) and choosing safe and qualified products are crucial for the prevention of rosacea. If makeup is necessary, simple medical make-up with foundation products should be recommended to minimize the risk to attack rosacea.

## Supporting information

S1 Data Questionnaire. (DOCX)

S1 File Rosacea work data. (SAV)
