# Supplementary material for: Effects of skin care habits on the development of rosacea: A multi-center retrospective case-control survey in Chinese population
Source: PLoS One. 2020 Apr 27;15(4):e0231078. doi: 10.1371/journal.pone.0231078 (PMC7185582; doi:10.1371/journal.pone.0231078)
Supplement: S1 Data — (DOCX) [file pone.0231078.s001.docx]

**Code** **_______**

**Questionnaire about skin care habits**

**Date：___________**

**Tel: ___________**

**Name________**

**□healthy control □patients with rosacea**

**Subtype of rosacea（only for rosacea patients）**

**□ETR**  **□PPR □PhR □Ocular rosacea**

**Demographic data**

1. You are male or female?

____________

2. How old are you?

____________

3.Would you tell me your education level?

#1 high school level or less;

#2 university or junior college level;

#3 master degree or above

4. Would you like to tell me your monthly income？

#1 less than 1000RMB;

#2 1000-2000RMB;

#3 2001-4000RMB

#4 more than 4000RMB

5. Do you have to work outdoors?

#1 Yes #2 No

**Skin Type**

**How do you feel about you skin nature?**

#1 greasy

#2 greasy T zone only

#3 dry

#4 without evident feature of oily or dry

**Skin type (evaluated by a dermatologist)**

#1 neutral;

#2 dry;

#3 oily;

#4 mixed;

**Skin care habits**

Please report the following skin habits in the past two years (if you are skin-healthy) or in the previous two years before the onset of rosacea (if you are a patient of rosacea)

1. How often did you take care of your skin?

#0 hardly;

#1 once a day;

#2 twice a day;

#3 three times or more a day

2. Did you use moisturizing products every day?

#1 Yes #2 No

3. Did you use any of the following functional products when you take care of your skin daily？(You may choose more than one options)

# Whitening; # anti-aging; # oil-control; # anti-allergy; # none of the above;

4. Usually, which way did you usually buy cosmetic products? (You may choose more than one options)

#0 cosmetic counter in the shopping mall/ pharmacy;

#1 beauty salon

#2 online shopping/ TV shopping

#3 DIY production

#4 Individual store or direct-selling

5. How often did you use a facial cleanser?

#1 hardly;

#2 1-3 times one week;

#3 almost every day or every other day;

#4 more than two times every day

6. What type of skin cleanser did you use most often?

# foaming cleanser;

# facial soup;

# non-foam type/ emulsion cleanser;

# deep cleansing or exfoliator

7. How often did you use a facial mask？

# hardly;

# about once a week;

# 2-3 times one week;

# 4 times or more every week

7. Did you go to the beauty salon for facial treatment regularly? If yes, how often did you go there?

#0 hardly;

#1 less than 2 times one month;

#2 3-4 times one month;

#3 more than once every week

8.What type of skincare projects did you usually do in beauty salon? (You may choose more than one options)

# moisturizing;

# whitening;

# anti-aging;

# oil-control;

# antiallergy;

# other

9. How often did you use sunscreen cream?

# hardly;

# 1-2 times one week;

# 3-5 times one week;

# almost every day,

10. How often did you make up ？

# hardly;

# 1-2 times one week;

# 3-5 times one week;

# almost every day,

11.The foundation products including makeup base, color correction cream, or blemish balm cream. So, did you use foundation products for makeup?

#1 hardly

#2 always

12. Did you use makeup remover products after makeup?

#1 hardly

#2 always

Thanks for answering!
